# Epistemic Trust and the Emergence of Conduct Problems: Aggression in the Service of Communication

**DOI:** 10.3389/fpsyt.2021.710011

**Published:** 2021-09-23

**Authors:** Alessandro Talia, Robbie Duschinsky, Diana Mazzarella, Sophie Hauschild, Svenja Taubner

**Affiliations:** ^1^Institute for Psychosocial Prevention, University of Heidelberg, Heidelberg, Germany; ^2^Primary Care Unit, University of Cambridge, Cambridge, United Kingdom; ^3^Cognitive Science Centre of the University of Neuchâtel, Neuchâtel, Switzerland

**Keywords:** attachment, language, aggression, epistemic trust, conduct problems

## Abstract

Fonagy and colleagues have recently proposed that deficits in the capacity for epistemic trust (i. e., the expectation that interpersonal communication is relevant to the addressee) are fundamental to psychopathology. In this paper, we consider the implications of this hypothesis for understanding the role of aggression in conduct disorder and conduct problems more generally. Our main proposal is to view conduct problems not only as reflecting dysregulation, but as an adaptation that allows communication with others who are (or are perceived to be) unreliable. Our formulation hinges on two propositions. The first one is to view aggression as a modality of communication adapted to scenarios in which the communicator expects the audience to have low epistemic trust in the communicator. The second idea is to conceptualize the failed “unlearning of aggression” as reflecting a lack of interest in maintaining one's reputation as a communicator, which in turn stems from a lack of epistemic trust in other communicators. In this paper, we discuss these ideas and examine how they may account for the developmental pathways that lead young people to develop conduct problems.

## Introduction

Conduct disorder is a global public health issue. It is diagnosed in children and adolescents who have a tendency to violate the rights of others and societal norms (1, 2), and it is associated with extremely high levels of service utilization and costs across the lifespan (3). It is the most common reason for referral to child and adolescent mental health services in developed countries [see e.g. (4, 5)], and its prevalence among juvenile offenders is in excess of 61% (6). Overall, conduct disorder accounts for ~1% of all years lived with disability at a global level (7).

While research has documented the diverse risk factors that predict conduct disorder [see (8)], integrating these into a comprehensive explanatory theory has proved to be more difficult. A major challenge in this direction is the heterogeneity of conduct disorder as a diagnostic category (9, 10). Around 40% of young patients with a diagnosis of conduct disorder engage in deliberate, goal-directed aggressive acts and rule-breaking, while the remaining 60% mainly tends to show reactive aggression in response to (perceived) threats (11). Some of these patients begin to experience conduct problems in early childhood, while others do so only in adolescence (12). Many have comorbid ADHD, while others experience severe mood and anxiety disorders in addition to conduct problems (13). Given such heterogeneity, doubts have been raised that we can identify a single causal explanation underpinning conduct disorder in all its different presentations (14). Several authors have suggested that, before we can indicate a specific core of this disorder, we will need to refine or subdivide the existing diagnosis further (8).

Such calls for greater specification encounter additional challenges. In fact, not only is conduct disorder internally heterogeneous, but it also has diffuse boundaries. Namely, it is far from clear how we can distinguish between children and adolescents who display antisocial behavior because of an individual dysfunction and those who primarily do so because of ongoing and sometimes short-lived environmental influences. It has been known for a long time that there are many children and adolescents who primarily display antisocial behavior as a reaction to negative environmental circumstances [see e.g., (15)], or because of group pressures toward modeling their deviant peers (16). During early toddlerhood and middle adolescence, an increase in conduct problems is even expected and considered “normal” (17). As a consequence, influential scholars have urged to make the existing criteria for diagnosing conduct disorder more stringent [e.g., (18)].

In this paper, we contribute to the evolving conceptualization of conduct disorder by exploring the meaning of aggression and rule-breaking, two behaviors that are at the core of the diagnosis (as well as other diagnoses, such as oppositional defiant disorder). Indeed, though further efforts to improve the diagnosis of conduct disorder are most welcome, its heterogeneity and fuzzy boundaries may suggest the need to consider alternatives to a disorder-centered approach to explanation [see (11)]. Our focus on conduct problems rather than on conduct disorder *per se* builds upon previous work by Fonagy and Luyten (11), who attempted to move past current diagnostic limitations of conduct disorder by examining its underlying mechanisms in a trans-diagnostic light.

In this paper we propose that conduct problems such as aggression and rule-breaking are at least in part the result of chronic or temporary failures in the capacity for *epistemic trust*. Following Fonagy and Allison (19), we define epistemic trust as the expectation that interpersonally communicated information is true and relevant^1^, and that communicators intend it to be so. Similar to these authors, we also adopt the view that a lack of epistemic trust may be a main factor undermining resilience to developing psychopathology in general. Our work here attempts to consider the implications of this hypothesis for understanding conduct problems in particular. Differently from previous approaches, we propose to view conduct problems as functional, if only sub-optimal, communicative adaptations, rather than merely as dysfunctions. Aggression, as we shall argue, seems to preserve the possibility of an individual to be listened to and understood when others have only minimal epistemic trust in him or her. The failure to inhibit aggressive behaviors and the tendency to violate rules, on the other hand, may reflect a disregard for protecting one's reputation when not cooperating seems beneficial, due to low epistemic trust in others.

A theory of conduct disorders based on epistemic trust should address a number of questions; in this paper, we will discuss them in turn. First, we will examine what factors may lead to decreased epistemic trust in the first place. Second, drawing from the work of Dan Sperber and other linguists, we will consider how diminished epistemic trust may determine distinct strategies for interpreting communication (21, 22). In the third and the fourth sections, basing ourselves on recent linguistics and evolutionary psychology, we will discuss our proposal of two mechanisms that underpin aggression and its failed inhibition. Though aggressive and rule-breaking behaviors are highly correlated (23, 24), we will suggest that the prevalence of the first or the second of the two mechanisms may explain the prominence of aggression or rule-breaking in different presentations of conduct disorder, respectively. Before outlining our new theory, we shall provide a short introduction to previous research on conduct disorder.

## A Brief Overview of Conduct Disorder

Conduct disorder is diagnosed, in both the DSM-5 and ICD-10, using a set of criteria that capture the consistent display of behaviors such as the tendency to assault people and animals, destroy other people's property, lie and deceive, and violate rules in general. Thus, unlike other mental disorders, a diagnosis of conduct disorder is generally made with exclusive reference to observed behavior, without attending to the subjective experience of the individual. For this reason, the symptoms that lead to a diagnosis of conduct disorder may be associated with varied underlying psychological experiences.

A first distinction must be made between presentations of conduct disorder that have an onset in childhood and those with an onset in adolescence (10). This is an important distinction, because the former subgroup—unless the disorder is limited to childhood—seems to be much more impacted in most life domains (25). A second distinction, emerging from factor-analytic studies, has identified two partly distinguishable subgroups of children: one characterized by reactive aggression and hypervigilance to others' emotional states; the other characterized by goal-oriented or instrumental violence and rule-breaking, rather than aggression *per se* [see (11)]. Individuals in this latter subgroup, which tend to overlap with the DMS-5 label “conduct disorder with limited prosocial emotions,” may demonstrate low empathy and callous behavior toward others. They seem to share a pool of substantially heritable traits (26), and seem to have more severe and persistent antisocial outcomes than the first sub-group (27, 28).

In the past decades, various models have emerged for explaining the development and mechanisms underlying conduct disorder. One of the most popular of these models emphasizes the role played by individual biases in social information-processing (29–31). According to this model, a tendency toward attributing hostile intentions to others may lead to reactive aggression [see e.g., (32)], while having positive expectations that aggressive behavior will lead to achieve instrumental goals may lead to rule-breaking and proactive aggression [see e.g., (33)].

While Dodge and colleagues' cognitive model proposes a plausible explanation of how aggression and rule-breaking may occur in any given situation, it does little in the way of explaining how the cognitive dysfunctions that are purported to cause them may develop [see (14)]. Various studies have tested different putative causes: increased amygdala reactivity to threatening facial expressions (34), early reinforcement of aggressive behavior (35, 36), and experiences of social rejection (37, 38). Crucially, following John Bowlby's pioneering intuitions (39), early insecure attachment relationships have been extensively documented in youths with conduct problems (40). Conduct problems have been found to be related to later antisocial behavior through the mediation of negative representations of relationships and coercive behavior (41).

A partly different theoretical approach for conceptualizing conduct disorder focuses not so much on the child's or adolescent's propensity for aggressive conduct, but on biological causes that may underpin children's *failure to inhibit* or *unlearn aggression* (42–45). It is commonly observed that, at least until the child's third year of life, most children frequently display aggression (46, 47). Thus, a key question to be addressed concerns what inhibitory processes may intervene in the majority of individuals, and why in certain individuals aggressive behavior persists and is consistently manifested in various domains.

In this respect, it is generally believed that aggression is inhibited when the individual is fully able to appreciate the consequences of one's actions on others' emotions. In this view, a primary flaw in encoding emotional stimuli would result in a failure to inhibit aggression. According to Blair (42), deficits in the threat response system located in the brain stem cause dysfunctions in recognizing other people's fear and distress. According to van Honk and Schutter (48), individuals with conduct disorder seem to share a pattern of low basic fearfulness, which determines reduced fear of punishment. Both frameworks propose that, especially in the case of conduct disorder with high callous-unemotional traits, these biological deficits may undermine the child's or adolescent's capacity for empathy and lead to decreased inhibition of aggressive behavior and rule-breaking.

Fonagy and his colleagues' early work on aggression and conduct problems can be seen as an attempt to identify a mechanism responsible for both the increased propensity for aggression, and the failure to inhibit it. At the roots of both, Fonagy and his colleagues saw a diminished ability to interpret the actions of others as motivated by intentional mental states, termed “mentalizing”. In this perspective, aggression is an effective way of temporarily getting rid of painful mental representations evoked by others, and of mentalizing as a whole (49, 50). Reciprocally, lower mentalizing impairs one's ability to consider the impact of one's actions on others, thus effectively nullifying the main reasons that are thought to inhibit aggression (51, 52).

## Factors That Influence Epistemic Trust

Our aim in this paper is to explore how conduct problems, such as aggression and rule-breaking, can be viewed as a consequence of low epistemic trust. In putting forward our proposal, we build upon recent work by Fonagy and his colleagues, who departed from their previous focus on impaired mentalizing as the main cause of psychopathology and increasingly underlined the role of epistemic trust (53, 54).

Fonagy and colleagues' emphasis on epistemic trust is inspired by several large (*N* > 20,000) longitudinal studies with psychiatric patients, which suggest that mental disorders in general are sequentially comorbid, recurrent/chronic, and can be aptly accounted for statistically by a single general factor of psychopathology called 'p factor' [see e.g., (55, 56)]. This is consistent with robust evidence from studies in behavioral genetics and molecular biology, which suggest that genetic risk for developing a mental disorder is not specific to any disorder in particular [see (57)]. Also taking into account that the most powerful predictor of mental illness is socioeconomic deprivation (58), Fonagy and his colleagues then argued that the “p factor” cannot be equated with a deficit in any single cognitive or affective skill—mentalisation or other. According to these authors, this general factor common to psychiatric disorders may be better conceptualized as a product of the relationship between individual dispositions and the social environment, or, briefly, as a lack of resilience within an individual's social system (53).

Fonagy and his colleagues tentatively linked such an absence of resilience to the inability to learn from others and being receptive to their support, which descends from low epistemic trust (54). They believe that, without epistemic trust, resilience in response to adversity is impaired. Young patients with conduct disorder, especially those with high callous-unemotional trait, can be viewed as an example of this tendency. They seem to show a poorer response to treatments based on social learning theory than other patients (59). Moreover, and perhaps adaptively, they seem less affected by harsh and coercive parenting in their later outcomes than other patients (60).

If, as we are persuaded, Fonagy and colleagues are correct in attributing a primary role—though perhaps not exclusive—to reduced epistemic trust in the development of psychopathology, this role needs considerable specification. Our goal in this paper is to sketch a preliminary framework for specifying the role of low epistemic trust in the instance of conduct problems. Our preliminary task in this section will be to discuss the concept of epistemic trust and what factors may generally determine its differing levels.

Factors that influence epistemic trust may be, on a first approximation, grouped into two classes: those that are context-specific; and those that reflect more general influences on how an individual engages in communication. The first set of factors include: the degree of the communicator's trustworthiness (e.g., speaker's expertise, past accuracy and benevolence, and access to relevant information), the strength of the evidence provided in support of the communicator's claims, and the consistency between that which is communicated and what was previously known by the addressee (61). This first set of factors have been thoroughly examined by philosophers interested in the epistemology of science and social communication [see e.g., (62)], and by empirical researchers on how infants learn from adults' testimony [see (63)]. These factors may play a key role whenever the individual has time to decide whether trust is justified.

Whenever such an informed evaluation is not possible, it is likely that epistemic trust is influenced to a greater extent by individual biases, and only to a lesser extent by context-sensitive judgments. Just by way of example, when others make conjectures about people's mental states, or when they share their subjective values and preferences, we can rarely draw on objective evidence that we can use to assess the accuracy of what they say (64). In these and other cases, our epistemic trust may be affected by fast-and-frugal heuristics geared to ascribe trust on the basis of our own trait-like dispositions to accord it or not (65).

It is remarkable that many or most of the factors that are linked to developing a mental disorder, including conduct disorder, can be also seen as influencing one's capacity for epistemic trust in a trait-like manner. For instance, impulsive or fearless temperaments, attentional dysfunctions, and low verbal IQ have all been associated with psychopathology in general and conduct disorder in particular (8). At the same time, it is likely that these factors, by making it more difficult to process communication at a cognitive level, may reduce children's trust in the relevance of interpersonal communication (i.e., may impair their capacity for epistemic trust).

Other early environmental influences known to be associated with psychopathology and conduct disorder may lead to reduced opportunities for relying on others' communication. Among these, we may mention maltreatment, insecure and disorganized infant-caregiver attachments, poverty and marginalization, low school readiness, and academic failure [see (57)]. Insecure and disorganized infant-caregiver attachments—which have been linked to increased psychopathology and externalizing problems (66)—may be an especially important influence on epistemic trust. Fonagy et al. have proposed that attachment relationships create an important context in which one develops epistemic trust (54). This hypothesis has recently been extended in order to re-conceptualize attachment-related differences as differences in epistemic trust [see (64, 67)].

## How Epistemic Mistrust Determines Problems in Interpersonal Communication

So far, we have discussed various factors that may exert an influence on epistemic trust. Our next step is to describe how low epistemic trust may underpin problems in communication, which in our view mediate the link between low epistemic trust and psychopathology in general, and conduct problems in particular.

It is intuitive enough to see that epistemic trust determines our tendency to accept a piece of information as true. Perhaps less intuitive, but equally important, is to realize that epistemic trust also seems to impact how we *interpret* the meaning of communication [see (21)]. Indeed, even before we decide whether to accept a piece of communicated information, we are confronted with the task of understanding what message communicators intends to convey through their verbal behavior. Linguistic pragmatics suggests that this task, carried out in an unconscious and spontaneous way, involves some measure of epistemic trust as well.

Human communication can rarely be viewed as a process of coding and decoding (20). Take, for instance, the sentence *You are not going to die*. It will acquire different meanings when stated by a lawyer to a prisoner fearing a death sentence, when proclaimed by a priest to the Sunday worshippers, or when uttered by a mother aiming to minimize her child's alarm for having cut his finger. In all of these scenarios, listeners are guided toward the correct interpretation by the words uttered, the context, and the expectation that the speaker intends to present his or her communication as relevant from the listener's point of view. The prisoner does not expect his lawyer to have lost her mind and announce to him his eternal life; the worshippers do not think they have literally conquered death; and the child knows that his mother means to tease him. All these various audiences are guided in their understanding by their epistemic trust in the speaker—in other words, by their assumption that the communication is true and relevant, and that the speaker intends it to be that way. In other words, whether or not the audience eventually accepts the communicated information as true, interpretation is already guided by some minimal stance of trust (68).

According to a popular theory in linguistic pragmatics, Relevance theory, this stance of trust in interpreting communication is triggered by any communicative behavior that seems overt and intentional, also termed *ostensive* (20). With this kind of behavior, the communicator does not simply communicate some information (as when, by wearing a tie, one covertly conveys an impression of distinction). Through ostensive behavior, the communicator also makes manifest an intention to communicate that piece of information. Seeing that communication is intentional, the listener presumes that the speaker aims to be relevant. According to this presumption, then, the listener goes on to interpret the speaker's communication, retrieving meanings that go beyond literal content, as in the examples above.

At this stage, we can anticipate two common problems that can arise in communication as a consequence of low epistemic trust. The first problem is that listeners with chronically low epistemic trust may interpret ostensive communication differently from what speakers expect them to (69). The second problem is that speakers themselves might already anticipate that listeners have low epistemic trust in them and forgo the use of ostensive communication altogether. Both problems are relevant to conduct disorder. We will examine them in turn, in this and in the next section, through a running example.

Imagine that you are sitting in a compartment of a train, heading home, next to an unknown traveling companion. You feel sleepy, and if you tilted your head you could no doubt doze off for a short nap. Unfortunately, your companion introduces himself and starts chattering about something inane. What could you do? A first possibility could be to pretend not to have understood that he is talking to you, not stifle a yawn and close your eyes. This communication is not ostensive. In this way, you would be producing *direct* evidence of your tiredness, and your recently met companion might stop bothering you.

An alternative possibility would be to say to your fellow traveler:

(1) “I am really tired; I didn't get any sleep yesterday.”

In other words, you can produce *indirect*, but nevertheless strong, evidence that you feel tired and therefore prefer not to talk. This is a case of ostensive communication. Consequently, not only you inform your listener that you are tired, but you also communicate your intention to inform him of this [see (20)]. As a consequence, and provided that the listener trusts the presumption of relevance communicated by you, he may understand your utterances as saying more than what you literally stated (e.g., he is likely to interpret your words as a way to stop the conversation, not simply as a disclosure of your tiredness). However, if your listener harbors doubt about your communicative competence or honesty (i.e., has low epistemic trust), these implicit meanings may not be derived. For instance, while he might interpret (4) as an invitation to interrupt the conversation, he may suspect that you are not tired and search for another more relevant interpretation (for instance, he might think that you are angry at him and react aggressively to your candid request).

Within the paradigm of attachment research, Talia et al. (64) have explored how insecure infant-caregiver attachment behavioral patterns may offer a typical example of low epistemic trust. Attachment patterns are assessed in the laboratory paradigm “Strange Situation” by observing how infants seek proximity toward the caregiver when under stress [SSP, (70)]. According to Talia et al.'s views, low proximity-seeking toward the caregiver (one of the main indicators of insecure infant-caregiver attachment) may be considered as reflecting a decreased interest in seeking information of any kind from the caregiver, including but not limited to support and reassurance.

Talia et al. have then proposed that, because adult communication and comprehension is based on epistemic trust, early attachment patterns may determine later differences in how individuals interpret and produce communication, termed *epistemic styles*. Epistemic styles are individual tendencies to focus attention onto aspects of communication that one unconsciously expects to be more trustworthy, in part as a consequence of early experiences with one's main attachment figures. Talia et al. have proposed that measures of attachment in adults, such as the Adult Attachment Interview (AAI) capture an instance of these broad epistemic styles, in the special case of discussing attachment experiences.

If this is correct, when compared to secure attachment classifications, insecure attachment classifications may generally reflect a more cautious, less trustworthy stance toward interpersonally communicated information, and be associated with cognitive biases in comprehension. This may explain why infants and adults who receive an insecure attachment classification tend to experience—with caregivers, partners, and peers—more interpersonal conflicts (71), are more inclined to expect that others have negative interpersonal intentions [see e.g., (72)], respond more negatively to moderate levels of interpersonal frustration [see e.g., (73)], and demonstrate a lesser capacity for repairing relational ruptures when these arise [e.g., (74)]. When considering conduct problems, such consequences of low epistemic trust seem especially salient and may suggest at least one specific mechanism linking insecure attachment with conduct problems.

## From Problems in Communication to Aggression

We have discussed so far how low epistemic trust influences how we interpret interpersonal communication. We now wish to propose, reciprocally, that the speaker's expectation about *the listener's* epistemic trust in him or her may influence how speakers unconsciously design their communication [a special case of *audience design*, i.e., the general phenomenon by which communicators design their messages by considering the perspective of the audience (75)].

We will say that, when a speaker does not expect to be trusted by his or her audience, aggression and violence are an effective way to supplement ostensive communication. With an audience who may not trust that the speaker intends to provide relevant information, aggression may be more immediately efficacious as a form of communication. To go back to our previous example, imagine that you are traveling companion is undeterred by your previous statement in (4) and keeps talking. You may then directly shut him up by imposing your hand on his mouth. Physical coercion—rather than an indirect request—requires considerably less cooperation from the listener, and whether or not the listener might trust you as a communicator is of negligible importance.

But there is another way to communicate in the absence of epistemic trust, which is less obvious than physical coercion, and was recently noted by Sperber in a specific example of animal communication:

[Communication] can become so obnoxious and the annoyance it causes so great that complying becomes desirable as a means to put an end to the insistence itself. A form of blackmail, so to speak: you satisfy my request, or I will keep pestering you. In such a case, however, compliance is achieved not by making the message more relevant and by convincing the audience of its legitimacy but by imposing a social cost on the addressee (76).

Returning to our example, let us imagine that you are not able to provide direct evidence of your tiredness, and you do not want to use physical force to stop your petulant interlocutor. At the same time, based on your past interaction, you do not expect from him any charitable interpretation of further ostensive communication. What to do? You might say:

(2) I can't stand all this f^******^ nonsense! Shush!

which you might utter out loud so as to be heard by the whole train car, prolonging your *shush* until your companion stops talking.

Insofar as aggression entails information transmission, it seems possible to view it as a type of communication. However, as in (5), aggression is a type of communication that is not, or not exclusively, ostensive. By uttering (5), you expect the listener to comply with your wish to end the conversation not in virtue of the recognition of your communicative intention, but as a way to minimize the cost that your behavior poses to him—that is, ongoing humiliation with other travelers or mere annoyance. We propose to term this type of communication *perseverative*. Perseverative communication does not work by creating an expectation that communication will be true and relevant to the audience, or high epistemic trust. It works by creating an expectation that not attributing informative value to the speaker's communication will be costly, cognitively or otherwise, and that the audience is better off complying for this reason^2^.

This perspective may account for why children classified as “disorganized” in the SSP [a main predictor of later externalizing behavior, (66)] tend to display controlling behavior after brief separations from their parents [see e.g., (77)]. As a classification, infant-caregiver disorganized attachment is based on the observation during the SSP of behaviors that suggest fear or conflict in relation to the caregiver. It is often considered an “extreme” version of attachment insecurity (78). In Main and Cassidy's study and its many replications (79), 6-year-old children who had been previously classified as “disorganized” in the SSP, after a 1-h-long separation from their main caregiver, were likely to display forms of communication that appear subtly controlling [see (77), p. 419]. Following Talia and colleagues' ideas discussed previously, we could view these controlling forms of communication as a consequence of extremely low epistemic trust in the caregiver. One could say that, in the impossibility to rely on ostensive communication as an effective way to modify the parent's thoughts and behaviors due to low epistemic trust, these children may be inclined to use perseverative communication instead. They seem to act in order to humiliate (“You're really clumsy”), embarrass (“I said, keep your eyes closed!”), or reject the parent (“don't bother me”), or to control the parent with over-bright or compulsively caregiving behavior (“want to play with me, mommy, in the sandbox? It's fun, isn't it, mummy?”).

The reliance on aggression, typical of conduct disorder, can be viewed as an attempt to inform the addressee when the addressee is perceived to have no epistemic trust in the communicator. The communicator may have from the start low expectation about others' epistemic trust, perhaps because of insecure or disorganized attachment experiences, low verbal IQ, or deficits in executive functioning of neurodevelopmental origin (all of which are known risk factors for developing conduct disorders). Or the behavior of another person, perceived as a slight or a provocation, may evoke aggressive behavior that unconsciously aims to increase the communicator's chances to be understood and believed.

All children experience moments in which they do not expect to be listened to or believed, and only some of them adopt aggressive behaviors in a repeated and stable manner. Two aspects should be mentioned here. The first is that ostensive communication is a developmental achievement. All young children wail, scream, and cry in order to attract and manipulate others' attention. Some children may be slower at picking up ostensive communication, and be more ready to use aggression in response to evidence of low epistemic trust in their addressees. A second aspect to consider is family dynamics, which are another important mediator for the influence of early experiences on later conduct problems. Work by Patterson et al. [see e.g., (80)] has shown how some children learn to avoid parental demands through coercive behavior in order to gain control over an unpleasant family environment. If the parent then disengages or withdraws in order to avoid conflict, this type of communication gradually becomes overlearned through negative reinforcement.

## From Aggression to Conduct Disorder

Not every individual who displays aggressive behavior early on goes on to develop conduct disorder. Many children may only display aversive behaviors in the family, but not exhibit similar behavior with other people in other settings, or they may never engage in violent behaviors such as physical aggression or stealing. That is, many children who do engage in aggressive behavior are able to successfully inhibit it in most contexts.

Understanding the process by which aggression is inhibited in toddlers may require gaining a better understanding of how moral conduct develops. We wish to refer here to a point of view on moral conduct termed “mutualistic” (81). According to this point of view, moral conduct is an adaptation to environments in which individuals compete to be recruited in mutually beneficial interactions [see e.g., (82, 83)]. Exploiting or ignoring others, even when in the short term might bring some benefits, eventually compromises one's own reputation and the possibility to be trusted by others. According to mutualistic theories of moral conduct, it is likely that humans have evolved an adapted propensity to judge the cost of behavior in terms of reputation.

Because computations of this sort may happen in part unconsciously and activate powerful affects such as shame or guilt, pro-social behavior is not exhibited only when one is observed by others (84). As Trivers suggested [(85). p. 51 cited in 66), “selection may favor distrusting those who perform altruistic acts without the emotional basis of generosity or guilt because the altruistic tendencies of such individuals may be less reliable in the future.”

The mutualistic perspective of moral conduct fits well with the special but fundamental case of communicative behavior. Because humans depend for their survival and well-being on the interpersonal transmission of information, any communication that risks compromising one's reputation as a communicator is dangerous. As a speaker, an individual will be more trustworthy if he or she refrains from using perseverative communication—such as threats and insults. The same can be said, of course, of speakers who misinform others, lie, and break their word. Reciprocally, an individual proves to be trustworthy if he or she manifests interest in what others intend to communicate, displays guilt or empathy, and pays close attention to social cues and the instructions given by others. By failing to do any of these things, an individual may alienate other communicators. Because children need to acquire a number of socio-cultural competencies before they are able to carry out such complex computations, one can make sense of the finding that aggression peaks at about age two and decreases only afterwards.

Individuals with conduct problems seem to engage in many behaviors that may compromise their reputation. Why? We hypothesize that a child or adolescent who has no epistemic trust that information communicated by others will be relevant may have “little to lose” if considered untrustworthy. Of course, an individual's epistemic trust will vary at different times and in different social groups. This means that aggression may be inhibited as a function of a child's or adolescent's differing expectations about the relevance of communication of others.

In this framework, the often observed linked between aggression and low mentalizing is a result of the fact that both of them descend from low epistemic trust. Contemporary approaches to pragmatics assume that mentalizing is heavily involved in language comprehension (86). When epistemic trust is high, mentalizing is guided by the assumption that the speaker has an intention to communicate relevant information. When epistemic trust is low—as when the subject exhibits aggressive behavior—the addressee may adopt different strategies for mentalizing communicative intentions, which might entail suspiciousness and hostile attributions.

It is important to underscore that many variables that we have not discussed here may be important in the development of conduct disorder. Temperament, gender, sexual maturity, and physical strength may enhance or limit one's potential for violence. Further, the most typical trajectories that lead to conduct disorders often include the encounter with a group of peers, which may provide positive reinforcement to the individual who displays violent behavior (87). Unmet needs for social bonding and acceptance might increase the appeal of such groups; among other things, they may provide a context in which the adolescent can experience epistemic trust.

## Discussion

In this article, we presented a theory that conduct disorders reflect a functional adaptation of communication geared to enhance communication when epistemic trust is low. We discussed how a lack of epistemic trust underlies significant problems in comprehension. We then made the proposal that aggression could be considered a strategy for communicating in situations in which the listener has low epistemic trust in the speaker. Finally, we argued that a lack of epistemic trust in interpersonal communication makes it less likely that aggressive behavior will be inhibited. The hypotheses presented here are an application of Fonagy and his colleagues' recent ideas on epistemic trust to the specific context of conduct problems. We hope that similar work may be conducted to apply the concept of epistemic trust to other serious mental disorders.

Given the speculative nature of our account, the hypotheses presented here are in need of further research and empirical testing. Our suggestion to consider aggressive behavior as a form of communication may be used to support research on its activating and deactivating mechanisms. Our notion of perseverative communication may foster a richer understanding of aggression within the framework of cognitive linguistics. Our proposal to view the inhibition of aggression in an evolutionary-based framework may encourage a productive exchange between the developmental psychopathology and mutualistic theories of moral conduct, which to our knowledge have not been in dialogue. Of course, in order to test these and other hypotheses, the recent attempts to develop reliable measures of epistemic trust are of key importance (88, 89).

We would be remiss not to acknowledge that our treatment of conduct problems was far from comprehensive. In particular, more work is needed for accounting for the influences of biological factors on epistemic trust. Although we view these influences as important, we did not examine them in depth because, at present, relevant empirical studies that may qualify this relationship are lacking.

We believe that the ideas presented in this theoretical article may have some practical implications. At least in part, our article concurs with some well-established views about the treatment of young people with conduct disorders. If conduct problems can be understood as resulting from a lack of epistemic trust in the relevance of communication, our ideas support the expectation that treatments for this condition may be most effective early on, when these young patients' interest in learning from other adults (e.g. the clinician) may still be partly open. Furthermore, because epistemic trust is a property of systems rather than individuals, our views seem to support the use of interventions acting at the family or peer-group level.

On the other hand, our approach may lead clinicians to potentially embrace new perspectives. Currently, treatments for conduct problems mainly focus on teaching parents how to enhance their children's impulse control, based on a social learning model [see e.g., (90)]. On the basis of our work, it seems possible to suggest that interventions aimed at fostering epistemic trust, through therapeutic work with the individual and their family, may be just as important. A common way in which clinicians already foster epistemic trust (regardless of whether they conceptualize their activity through this concept) is helping create a collaborative treatment relationship in which the patient can trust clinicians' communications and learn to mentalize (19). Another way, even more germane to the core proposal of this paper, draws from the idea that epistemic trust also influences how a speaker *produces* communication. Our focus seems to encourage therapeutic work on *how* young patients and their families communicate, rather than exclusively on the content of their communication. In our perspective, the task of clinicians may be to show that *they* trust the patient, that they take his or her perspective seriously, and that ostensive communication can foster mutual understanding and connection (91).

## Data Availability Statement

The original contributions presented in the study are included in the article/supplementary material, further inquiries can be directed to the corresponding author/s.

## Author Contributions

AT elaborated the original theoretical proposal presented in this article and wrote the first draft. All authors revised, edited, and expanded the manuscript and are responsible for its final version.

## Funding

This work was supported in part by the International Psychoanalytic Association (Grant number 055).

## Conflict of Interest

The authors declare that the research was conducted in the absence of any commercial or financial relationships that could be construed as a potential conflict of interest.

## Publisher's Note

All claims expressed in this article are solely those of the authors and do not necessarily represent those of their affiliated organizations, or those of the publisher, the editors and the reviewers. Any product that may be evaluated in this article, or claim that may be made by its manufacturer, is not guaranteed or endorsed by the publisher.

## Footnotes

^1^The notion of relevance used in this paper is drawn from Relevance theory (20). In Relevance theory, a piece of communicated information is relevant, among other things, if it is possible to derive true consequences from it. In this use of the concept, “relevance” entertains a relation of entailment with truth.

^2^As noted by Sperber (76), ostensive and (to use our terminology) perseverative communication may co-occur. For example, aggressive behaviors such as threats and offenses seem to achieve relevance in both ways simultaneously. They communicate some thoughts of the speaker, as well as the speaker's intention to communicate those thoughts. However, until the threat has been withdrawn, or the offense erased, a threat or an insult continues to act as a way to coerce the addressee by inflicting a social cost. Physical aggression, too, can be seen as communication that partly relies on ostension, partly on insistence. Merely imposing my hand over someone's mouth is probably insufficient to coerce any but the physically weakest interlocutor. What makes my gesture an effective way to stop someone from speaking is their unconscious recognition of my intention to impose on them an increasingly high cost until they comply with my request.
